# Microglial Activation After Systemic Stimulation With Lipopolysaccharide and *Escherichia coli*

**DOI:** 10.3389/fncel.2018.00110

**Published:** 2018-04-24

**Authors:** Inge C. M. Hoogland, Dunja Westhoff, Joo-Yeon Engelen-Lee, Jeroen Melief, Mercedes Valls Serón, Judith H. M. P. Houben-Weerts, Inge Huitinga, David J. van Westerloo, Tom van der Poll, Willem A. van Gool, Diederik van de Beek

**Affiliations:** ^1^Department of Neurology, Center of Infection and Immunity Amsterdam, Academic Medical Center, University of Amsterdam, Amsterdam, Netherlands; ^2^Netherlands Institute for Neuroscience - KNAW, Amsterdam, Netherlands; ^3^Intensive Care Medicine, Leiden University Medical Center, Leiden, Netherlands; ^4^Center of Experimental Molecular Medicine, Academic Medical Center, Amsterdam, Netherlands

**Keywords:** microglia, microglial activation, systemic infection, lipopolysaccharide, *Escherichia coli*, neuro-inflammation, mouse model

## Abstract

**Background:** Microglial activation after systemic infection has been suggested to mediate sepsis-associated delirium. A systematic review of animal studies suggested distinct differences between microglial activation after systemic challenge with live bacteria and lipopolysaccharide (LPS). Here, we describe a mouse model of microglial activation after systemic challenge with live *Escherichia coli* (*E. coli*) and compare results with systemic challenge with LPS.

**Methods:** Sixty mice were intraperitoneally injected with *E. coli* (1 × 10^4^ colony-forming units) and sacrificed at 12, 20, 48, and 72 h after inoculation. For 48 and 72 h time points, mice were treated with ceftriaxone. Thirty mice were intraperitoneally injected with LPS (5 mg/kg) and sacrificed 3 and 48 h after inoculation; 48 control mice were intraperitoneally injected with isotonic saline. Microglial response was monitored by immunohistochemical staining with Iba-1 antibody and flow cytometry; and inflammatory response by mRNA expression of pro- and anti-inflammatory mediators.

**Results:** Mice infected with live *E. coli* showed microglial activation 72 h post-inoculation, with increased cell number in cortex (*p* = 0.0002), hippocampus (*p* = 0.003), and thalamus (*p* = 0.0001), but not in the caudate nucleus/putamen (*p* = 0.33), as compared to controls. At 72 h, flow cytometry of microglia from *E. coli* infected mice showed increased cell size (*p* = 0.03) and CD45 expression (*p* = 0.03), but no increase in CD11b expression, and no differences in brain mRNA expression of inflammatory mediators as compared to controls. In mice with systemic LPS stimulation, microglial cells were morphologically activated at the 48 h time point with increased cell numbers in cortex (*p* = 0.002), hippocampus (*p* = 0.0003), thalamus (*p* = 0.007), and caudate nucleus/putamen (*p* < 0.0001), as compared to controls. At 48 h, flow cytometry of microglia from LPS stimulated mice showed increased cell size (*p* = 0.03), CD45 (*p* = 0.03), and CD11b (*p* = 0.04) expression. Brain mRNA expression of TNF-α (*p* = 0.02), IL-1β (*p* = 0.02), and MCP-1 (*p* = 0.03) were increased as compared to controls.

**Interpretation:** Systemic challenge with live *E. coli* causes a neuro-inflammatory response, but this response occurs at a later time point and is less vigorous as compared to LPS stimulation.The *E. coli* model mimics the clinical situation of infection associated delirium more closely than stimulation with supra-natural LPS.

## Introduction

A delirium is the most common complication among hospitalized older people and has been associated with detrimental long-term effects (Inouye, 2006). A meta-analysis showed that delirium is associated with long-term risk of death, institutionalization, and dementia, independent of important confounders (Witlox et al., 2010). It is generally known that a peripheral infection is a common cause of delirium; however, the pathogenesis is still poorly understood. Evidence from animal and human studies suggests that microglial cells play a crucial role (Lemstra et al., 2007; Cunningham, 2013).

Microglial cells, the tissue macrophages of the central nervous system (CNS), are considered to be the main cell type of the innate immune system in the CNS. In a normal situation, microglia are regulated tightly in a balanced environment of pro- and anti-inflammatory mediators produced by surrounding healthy brain tissue. Threats to the homeostasis of the CNS, for example a peripheral infection, can provoke a rapid change in phenotype of the microglia from surveilling cells into active cells producing inflammatory mediators. In high concentrations, these inflammatory mediators are potentially neurotoxic and activate other surveilling microglia. Normally, microglial activation is regulated by inhibitory mechanisms so this vicious circle becomes interrupted. Examples of known inhibitory mechanisms on microglial cells are the cholinergic anti-inflammatory pathway (Shytle et al., 2004), fractalkine (CX3CL1) and its receptor (CX3CR1) (Cardona et al., 2006), cluster of differentiation 200 (CD200) and its receptor (CD200R) (Hoek et al., 2000), and cluster of differentiation 47 (CD47) and its receptor signal regulatory protein α (SIRP-α) (Oldenborg et al., 2001). A possible explanation for the association of delirium with poor outcome is that activated microglia escape their inhibitory mechanisms, fueling chronic uncontrolled neuro-inflammation (Witlox et al., 2010). To test this hypothesis there is a need for a clinical relevant animal model.

In a systematic review of the literature we described that, in experimental studies in rodents, peripheral challenge with lipopolysaccharide (LPS) causes a profound immunological response in the brain resulting in microglial activation (Hoogland et al., 2015). However, this LPS model poorly simulates the clinical situation, where inflammation is normally caused by infection with live bacteria and is commonly treated with antibiotics. In literature, studies examining microglial activation after systemic challenge with live bacteria are scarce, but results suggest that live bacteria cause microglial activation that is less profound as compared to that found in the experiments using a challenge with LPS.

The main purpose of this study is to introduce a mouse model where systemic challenge with live bacteria causes a neuro-inflammatory response. Secondly, we want to illustrate the differences in course of neuro-inflammation between systemic challenge with live bacteria and systemic challenge with LPS. We intraperitoneally injected mice with live *E. coli* or with LPS. Microglial response was measured using immunohistochemical staining with ionized calcium-binding adaptor molecule 1 (Iba-1) antibody (cell number and morphology) and flow cytometry (cell size, CD45 and CD11b expression); and inflammatory response by measuring messenger ribonucleic acid (mRNA) expression of pro- and anti-inflammatory mediators.

## Materials and methods

### Animals

In total, 138 specific pathogen-free 8-week old male wild type C57BL/6J mice were purchased from Charles River (Maastricht, The Netherlands) and maintained at the animal care facility of the Academic Medical Centre (University of Amsterdam). Mice were housed in groups of 3 to 6, in individually ventilated cages (IVC), for at least 2 weeks before testing. Ambient temperature was 19–24°C with 40–70% humidity. According to national guidelines, food and water were available *ad libitum* and a 12:12 h light-dark cycle was retained. All experiments were approved by the Institutional Animal Care and Use Committee of the Academic Medical Center (Amsterdam, the Netherlands).

### Bacteria

*E. coli* (K1:O18) was grown to an optical density at 600 nm in Luria-Bertani medium (LB) at 37°C corresponding to midlog phase. *E. coli* were harvested by centrifugation at 3,000 rpm for 10 min and washed twice with pyrogen-free sterile isotonic saline. Bacteria were diluted to a final concentration of 1 × 10^4^ colony-forming units (CFU's) in 200 μl pyrogen-free sterile isotonic saline. Serial 10-fold dilutions of the final bacterial inoculum were plated on blood agar plates and incubated overnight at 37°C to verify the amount of viable bacteria injected.

### Experimental procedures

In experiments investigating the effects of a bacterial infection, mice (*n* = 15 per time point) were given a single intraperitoneal injection of 1 × 10^4^ CFU in 200 μl pyrogen-free sterile isotonic saline (range 1.0 × 10^4^−1.8 × 10^4^ CFU). Control mice (*n* = 6 per time point) received 200 μl of pyrogen-free sterile isotonic saline via intraperitoneal injection. Inoculations were performed in different experiments and mice were sacrificed at 12, 20, 48, and 72 h after inoculation. As the administered dose of *E. coli* is lethal after 20–22 h, we treated the mice that were sacrificed 48 and 72 h after inoculation with ceftriaxone. Ceftriaxone (Fresenius Kabi, Den Bosch, the Netherlands) was intraperitoneally injected 12 and 24 h after *E. coli* injection in a dose of 20 mg/kg. For these time points (48 and 72 h) an extra control group (*n* = 6 per time point) with ceftriaxone treatment was added. Mice that were sacrificed 12 h after inoculation were only used for analysis of sickness behavior, weight loss and bacterial outgrowth to explore the *E. coli* infected mouse model.

In experiments investigating the effects of LPS injections, mice (*n* = 15 per time point) received LPS derived from *E. coli* (O111:B4 strain, Ultrapure, Invivogen) at a dose of 5 mg/kg via intraperitoneal injection. This dose and strain was chosen for its reliable induction of microglia activation and reproducible cytokine response in the brain (Hoogland et al., 2015). Control mice (*n* = 6 per time point) received 200 μl of pyrogen-free sterile isotonic saline via intraperitoneal injection. Mice were sacrificed at 3 and 48 h after LPS or pyrogen-free sterile isotonic saline injection.

Inoculation for all experiments was done at the same time of day, around 8.00 a.m. For the LPS model we chose the most commonly used dose and strain for LPS in literature; 5 mg/kg of *E. coli* O111:B4. For that reason we chose to use *E. coli* for the live bacteria model, but due to technical limitations we used a different strain, K1:O18.

Time points for collecting tissues differ between LPS injected mice (*t* = 3 h and *t* = 48 h) and for *E. coli* injected mice (*t* = 20 h, *t* = 48 h, and *t* = 72 h). Hence, direct comparison of results between LPS injected mice with those injected with *E. coli* is not possible. These time points were chosen based on available literature, which shows neuro-inflammatory response to LPS injection after approximately 3 to 6 h, and moderate neuro-inflammatory response after injection of live bacteria after roughly 1 to 7 days (Hoogland et al., 2015). Therefore, we chose different time points to examine early response for the two groups. Additionally, based on this literature, we expected more profound neuro-inflammatory response after injection of live bacteria to occur even later and included the 72 h time point for this group.

The weight of all mice was measured before inoculation, administration of ceftriaxone, or saline and mice termination. Weight loss was considered a quantitative measurement for the degree of sickness. Additionally sickness behavior was monitored during experiments. The design of the different experiments is illustrated in Appendix Table 1.

Mice were anesthetized by intraperitoneal injection of ketamine (190 mg/kg, Eurovet Animal Health, Bladel, the Netherlands) and medetomidine (0.3 mg/kg, Pfizer Animal Health, Capelle aan den IJssel, the Netherlands). Body weight was assessed, after which cardiac puncture for blood collection followed. Blood was collected in sterile tubes containing EDTA and stored on ice. The abdomen was opened and the vena cava was severed, subsequently cerebral spinal fluid (CSF) was collected by puncture of the cisterna magna, collected in sterile tubes and stored on ice. Thereafter the thorax was opened and perfusion of organs with sterile phosphate buffered saline (PBS) was performed via the left cardiac ventricle (approximately 20 ml PBS in 5 min). Next the spleen, the median lobe of the liver and the brain were harvested. The right hemisphere was suspended in 10% buffered formalin and embedded in paraffin for histopathology. The left hemisphere was either suspended in 5 ml of Hibernate-A medium (Invitrogen) and stored by 4°C (*n* = 5 experimental group/*n* = 3 control group) or taken up in 20% weight per volume sterile saline (*n* = 10 experimental group/*n* = 3 control group) as well as the median lobe of the liver and the spleen. The organs suspended in sterile saline were put on ice and were disrupted with a tissue homogenizer. Directly after homogenizing the organs, 50 μl of the tissue homogenate was suspended in 350 μl RA1 lysis buffer (Kit content of NucleoSpin® RNAII, Macherey-Nagel) and stored at −80°C for mRNA isolation. CSF was diluted 1:100 in sterile saline because of the low collected volumes. Serial 10-fold dilutions of blood, CSF, liver, and spleen homogenates were plated on blood agar plates and bacteria were allowed to grow overnight at 37°C.

### Isolating microglia for flow cytometry

After overnight storage in Hybernate-A medium at 4°C, left hemispheres (approximately weighing 250 mg) were meshed through a 70 μm cell strainer (Nylon, BD Falcon) in a glucose-potassium-sodium buffer [GKN-BSA; 8 g/l NaCl, 0.4 g/l KCl, 1.77 g/l Na_2_HPO_4_.2H_2_O, 0.69 g/l NaH_2_PO_4_.H_2_O, 2 g/l D-(1)-glucose, pH 7.4] with 0.3% bovine serum albumin (Roche) and collected in 50 ml tubes. After centrifuging (1400 rpm, 7 min, 4°C), cell pellets were suspended in 1 ml enzyme buffer (4 g/l MgCl_2_, 2.55 g/l CaCl_2_, 3.73 g/l KCl, and 8.95 g/l NaCl, pH 6–7), followed by enzymatic digestion in collagenase type I (370 units, Worthington) and DNase I (10 mg/ml, Roche) for 45 min, at 37°C, while shaking. After enzymatic dissociation, cells were washed with GKN-BSA buffer and incubated for 2 min on ice in 2 ml cold erythrocyte lysis buffer (8.3 g/l NH_4_Cl, 1 g/l KHCO_3_, and 0.03 g/l EDTA, pH 7.4). Subsequently, cells were washed and resuspended in 20 ml Percoll (GE healthcare) of ρ = 1.03, then underlaid with 10 ml Percoll of ρ = 1.095 and overlaid with 5 ml of GKN-BSA buffer. The tubes were centrifuged for 35 min at 1200 × g at 20°C, with slow acceleration and no break. The myelin layer on the top of the ρ = 1.03 phase were discarded and cells were collected from the interface between ρ = 1.095 and ρ = 1.03 Percoll. Next cells was washed and counted with a Coulter counter (Beckman Coulter, Z2). Approximately 1 × 10^5^ cells from every sample were transferred into separate polystyrene coated round bottomed 5 ml tubes (BD Falcon). Depending on cell numbers, a portion of every sample was put in a pool tube, which subsequently was divided over 5 tubes for a blanc sample and single stainings, every tube contained approximately 1 × 10^5^ cells.

Cells were stained in a total volume of 200 μl using antibodies with the following specificities: rat anti-mouse CD11b (immunoglobulin (Ig)G2b κ, clone M1/70, labeled with phycoerythrin (PE), 1:200, BD Pharmigen, rat anti-mouse CD45 [IgG2b κ, clone 30-F11, labeled with allophycocyanin (APC), 1:500, eBioscience]. To block aspecific binding normal mouse serum (1:10) and anti-mouse CD16/CD32 (Alias Fcγ III/II receptor, IgG2b κ, clone 2.4G2, 1:100, BD Pharmigen) was added. Cells were incubated with antibodies for 30 min on ice in polystyrene coated round bottomed 5 ml tubes. About 10 min prior to fixation, 2.5 μl of 7-amino-actinomycin D (7-AAD, labeled with peridinin-chlorophyll-protein (PerCP), 1:80 concentration, BD Pharmigen) was added per sample. After staining, cells were washed and fixated in 2% paraformaldehyde for 10 min on ice. Cells were washed and resuspended in 200 μl GKN-BSA buffer. Flow cytometric analysis was performed on a FACSCalibur machine (BD) and data were analyzed using FlowJo software version 7.6.1.

### Immunohistochemistry

Paraffin-embedded brain tissue was sectioned at a thickness of 5 μm in a coronal plane from the olfactory bulb to the beginning of the cerebellum. Starting from 1400 μm, sections were selected at intervals of 500 μm throughout the whole brain and mounted on slides (12 sections per mouse). After removal of paraffin in xylene and rehydration in a graded series of alcohols (100, 100, 95%), sections were incubated for 20 min in 0.3% hydrogen peroxidase (H_2_O_2_) diluted in methanol to block the endogenous peroxidase activity. Antigen retrieval was performed by incubation for 10 min at 120°C in 0.01 M citrate buffer (pH 6.0). Slides were washed with PBS and incubated with the primary antibody; Iba-1 (rabbit polyclonal, 1:2000, Wako Pure Chemical Industries). Slides were incubated with Iba-1 for 1 h at room temperature. Thereafter, sections were washed with PBS and were incubated for 30 min at room temperature with the ready-for-use PowerVision peroxidise system (HRP, Immunologic). As a chromogen 3.3′-diaminobenzidine (DAB, Sigma) was used and sections were counterstained with hematoxylin (1:10 diluted, incubated for 3 min).

Luxol fast blue—Periodic acid-Schiff—hematoxylin staining was used to discriminate between white and gray matter and was compared to microglial staining to select different brain regions for analysis. In the end slides were dehydrated and coverslipped with Pertex.

### Quantification of immunohistochemistry images

After staining all slides were scanned with a D. Sight fluo (A. Menarini, Florence, Italy) at 20x magnification. Two square millimeter of each digital image of cortex, hippocampus, thalamus, and caudate nucleus/putamen was selected. Microglial cell bodies were manually counted and morphologically reviewed by one observer, who was blinded for all mice characteristics (ICMH).

### Definition of microglial activation

Microglial cells were defined as activated based on the following criteria: (1) microglia showed an activated morphology based on immunohistochemical staining; (2) there was a significant increase in microglial cell number, quantified with immunohistochemical staining, (3) there was a significant increase in size of microglia, quantified with flow cytometry (increase in forward scatter compared to the control group); and (4) there was a significant increase in expression of a microglial marker, quantified with flow cytometry (expression of CD45 and/or CD11b). When 3 or 4 of the stated parameters were positive, microglial cells were defined as activated. When 1 or 2 parameters were positive, microglial cells were defined as moderately activated and when all parameters were negative, microglial cells were defined as inactive.

### RNA extraction and real time qPCR

Total ribonucleic acid (RNA) was extracted from murine brain and spleen homogenates using the nucleospin II extraction kit (Macharey-Nagel GmbH, Duren, Germany). The concentration of the RNA was measured using Nanodrop Spectophotometer (Nanodrop Technologies, USA) and the purity was assessed by the ratio of absorbance at 260 and 280 nm. RNA purity was within range of 2.0–2.1. Complementary deoxyribonucleic acid (cDNA) was synthesized from equal amounts of RNA using Iscript^tm^ according to the manufacturer's protocol (Biorad Laboratories, Hercules, USA). Gene-specific analysis by real-time quantitative polymerase chain reaction (qPCR) was performed using an iCycler MyiQTM system with Bio-Rad iQ^tm^SYBRGreenSupermix (Biorad Laboratories, Hercules, USA). Expression levels were normalized to reference gene Non-POU-domain containing octamer binding protein (NoNo). Primer sequences are depicted in Appendix Table 2. A negative control without the Reverse Transcriptase was also used. Data were analyzed using the Bio-Rad MyiQ Optical system Software version 1.0 and expression data were calculated using the delta cycle threshold (deltaCt) method.

### Statistical analysis

Continuous variables were tested with Mann–Whitney *U*-tests or Student *t*-tests depending on sample size and distribution of the data. The assumption of normality was tested with the Kolmogrov-Smirnov test. Since time points, type of infection and treatment were variable, we only compared an infected group with its control group and did not directly compare the different time points or infected groups with one another. Statistical significance was set to *P* ≤ 0.05. Because of the exploratory nature of this study, correction for multiple comparisons was not applied. For visual presentation of quantitative results, GraphPad Prism was used (GraphPad Software, version 6.07, La Jolla, CA, USA).

## Results

### Description of mouse models

To delineate differences of microglial activation after systemic stimulation with live *E. coli* and LPS, mice were either intraperitoneally injected with *E. coli* (1 × 10^4^ CFUs) and sacrificed at 12, 20, 48, and 72 h or with LPS (5 mg/kg) and sacrificed after 3 and 48 h; *E. coli* infected mice were treated with intraperitoneal injection with ceftriaxone (48 and 72 h time points). At 20 and 24 h, all mice showed signs of sickness; which consisted of reduction of movement and grooming. Behavior returned to normal at 48 h although the mean weight loss was 5% (95% confidence interval [CI] 3–7%) in the *E. coli* group and 7% ([CI] 5–9%) in the LPS-group (Appendix Figure 1). CFUs in *E. coli* infected mice at the 12 h time point were elevated for blood, spleen, and liver, but not for (CSF). At 20 h after *E.coli* injection CFUs were elevated for blood, spleen, liver, and CSF. CFUs decreased after ceftriaxone treatment; CSF cultures of all *E. coli* mice were negative at 48 and 72 h after infection (Appendix Figure 2).

### Microglial activation

**Figure 1:**
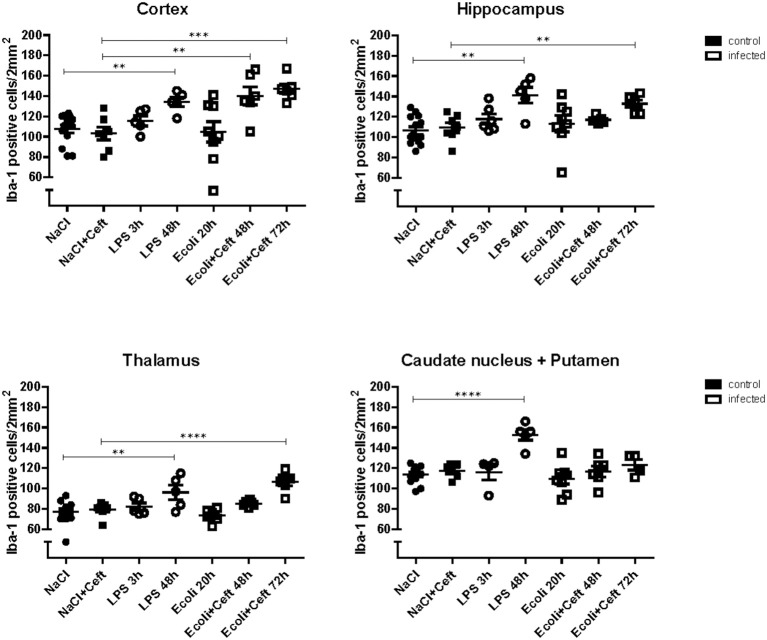
Histopathological counts of microglia. Number of Iba-1 positive microglial cell bodies per group (NaCl: *n* = 13, NaCl+Ceft: *n* = 7, LPS 3 h: *n* = 5, LPS 48 h: *n* = 5, *E. coli* 20 h: *n* = 8, *E. coli*+Ceft 48 h: *n* = 6, *E. coli*+Ceft 72 h: *n* = 6), in different brain regions (cortex, hippocampus, thalamus, and caudate nucleus+putamen), per 2 mm^2^.Data represent mean ± SEM, **P ≤ 0.01, ***P ≤ 0.001, ****P ≤ 0.0001.

**Figure 2:**
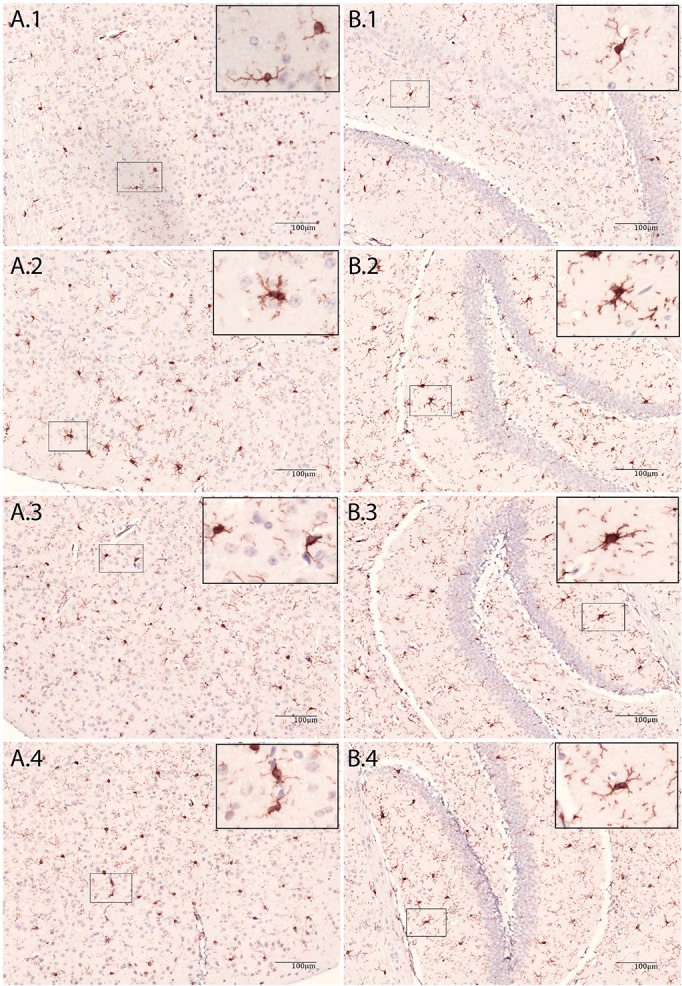
Immunohistochemic staining of brain tissue with Iba-1 antibody. **(A)** Cortex, **(B)** Hippocampus, 1: NaCl, 2: LPS 48 h, 3: *E.coli* + Ceftriaxone 48 h and 4: *E.coli* + Ceftriaxone 72 h.

**Figure 3:**
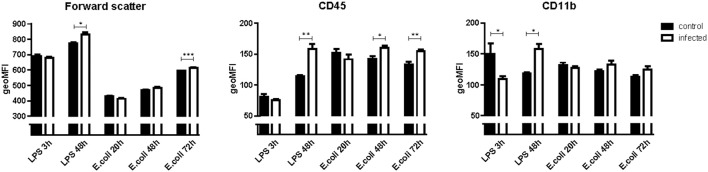
Geometric means (geoMFI) for forward scatter, expression of cluster of differentiation 45 (CD45) and CD11b measured with flow cytometry. Data represent mean ± SEM, *P ≤ 0.05, **P ≤ 0.01, ***P ≤ 0.001. NB: Flow cytometry for every time point was done on a different day. Laser characteristics vary per day, therefore the geoMFI'S are not comparable between experiments. Hence every infected group (*n* = 5) has its own control group (*n* = 3).

In *E. coli* infected mice, microglia activation parameters were negative at the 20 h time point post-stimulation. At 48 h, immunohistochemistry showed increased cell number in cortex only (mean 140 cells/2 mm^2^ [95% CI 117–163 cells/2 mm^2^] vs. 103 cells/2 mm^2^ [CI 88–118 cells/2 mm^2^] controls; *p* = 0.005), but no differences in hippocampus (mean 117 cells/2 mm^2^ [CI 112–122] vs. 109 cells/2 mm^2^ [CI 97–122]; *p* = 0.24), thalamus (mean 85 cells/2 mm^2^ [CI 82–88] vs. 79 [73–86]; *p* = 0.10), and caudate nucleus/putamen (mean 117 cells/2 mm^2^ [CI 103–131] vs. 118 cells/2 mm^2^ [CI 110–125]; *p* = 0.94; Figure 1). There were no morphological changes on immunohistochemistry at the 20 and 48 h time points (Figure 2). At 48 h, flow cytometry showed increased CD45 expression (*p* = 0.04), but similar cell size (*p* = 0.15) and CD11b expression (*p* = 0.24), as compared to isolated microglia cells from our control mice (Figure 3). However, at 72 h, immunohistochemistry showed increased cell number in cortex (mean 147 cells/2 mm^2^ [CI 135–159] vs. 103 cells/2 mm^2^ [CI 88–118]; *p* = 0.0002), hippocampus (mean 133 cells/2 mm^2^ [CI 124–142] vs. 109 cells/2 mm^2^ [CI 97–122]; *p* = 0.003), and thalamus (mean 107 cells/2 mm^2^ [CI 96–117] vs. 79 cells/2 mm^2^ [CI 73–86]; *p* = 0.0001), but not in the caudate nucleus/putamen (mean 123 cells/2mm^2^ [CI 107–140] vs. 118 [CI 110–125]; *p* = 0.33; Figure 1), and moderate morphological activation (Figure 2). Consistent with this finding, flow cytometry at 72 h showed increased cell size (*p* = 0.03) and CD45 expression (*p* = 0.03), without changes in CD11b expression (Figure 3).

In LPS injected mice, microglia activation parameters were negative at the 3 h time point post-stimulation. However, at 48 h, immunohistochemistry showed increased cell number in cortex (mean 134 cells/2 mm^2^ [CI 121–147] vs. 108 cells/2 mm^2^ [CI 99–116] controls; *p* = 0.002), hippocampus (mean 141 cells/2 mm^2^ [CI 120–163] vs. 106 cells/2 mm^2^ [CI 99–114]; *p* = 0.0003), thalamus (mean 96 cells/2 mm^2^ [CI 76–116] vs. 77 cells/2 mm^2^ [CI 72–83]; *p* = 0.007), and caudate nucleus/putamen (mean 153 cells/2 mm^2^ [CI 138–167] vs. 114 cells/2 mm^2^ [CI 109–119]; *p* < 0.0001; Figure 1). Additionally, microglial cells showed morphological activation (Figure 2) and flow cytometry of isolated microglial cells showed increased cell size (*p* = 0.02), and expression of CD45 (*p* = 0.04) and CD11b (*p* = 0.04) at 48 h (Figure 3).

### Inflammatory mediators

**Figure 4:**
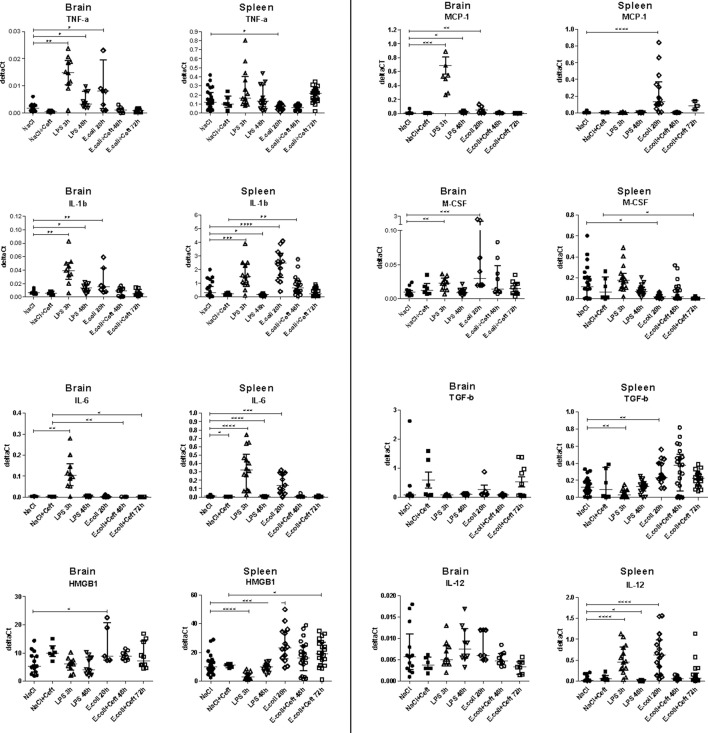
mRNA expression of TNF-α, IL-1β, IL-6, HMGB1, MCP-1, M-CSF, IL-12, and TGF-β illustrated in deltaCt's for brain, spleen, and liver homogenate. Data represent median ± IQR, *P ≤ 0.05, **P ≤ 0.01, ***P ≤ 0.001, ****P ≤ 0.0001.

We investigated inflammatory response in brain and spleen homogenate by measuring mRNA expression of pro-inflammatory mediators tumor necrosis factor alpha (TNF-α), interleukin 1 beta (IL-1β), interleukin 6 (IL-6), interleukin (IL-12) and high-mobility group 1 (HMGB1), immune regulators monocyte chemotactic protein 1 (MCP-1), macrophage colony-stimulating factor (M-CSF), and anti-inflammatory mediator transforming growth factor beta (TGF-β) (Figure 4).

In *E. coli* infected mice, mRNA expression of TNF-α, IL-1β, HMGB1, and M-CSF in brain homogenate was increased at the 20 h time point as compared to controls. At 48 and 72 h, all expression levels were similar between *E. coli* infected and control groups. In spleen, mRNA expression of IL-1β, IL-6, IL-12, HMBG1, MCP-1, and TGF-β was significantly increased 20 h post-infection, while expression of M-CSF was significantly decreased. At 48 h, mRNA expression of all expression levels turned to that of the controls, except for IL-1β (significant increase), while at 72 h expression of HMGB1 was significantly increased and M-CSF was significantly decreased.

In LPS injected mice, brain mRNA expression levels of TNF-α, IL-1β, IL-6, MCP-1, and M-CSF in brain homogenates were elevated at the 3 h time point as compared to controls. At 48 h, expression of TNF-α, IL-1β, and MCP-1 remained elevated to some extent, while other brain expression levels returned to that of controls. In spleen homogenate, the systemic compartment, mRNA expression of IL-1β, IL-6, IL-12 was significantly increased at the 3 h time point, while expression of HMGB1 and TGF-β was significantly decreased. At 48 h, mRNA expression was significantly decreased for IL-1β, IL-6, and IL-12 as compared to the controls and HMGB1 expression remained relatively low.

### Effect of ceftriaxone

Because a lethal dose of *E. coli* was used, mice that were sacrificed 48 and 72 h after inoculation were treated with ceftriaxone. To monitor the effect of ceftriaxone we included two extra control groups. Ceftriaxone or pyrogen-free sterile isotonic saline was intraperitoneally injected 12 and 24 h after pyrogen-free sterile isotonic saline injection. Immunohistochemistry, flow cytometry, and mRNA expression of pro-inflammatory mediators did not differ between these control groups (respectively Figure 1; Appendix Figure 3; and Figure 4).

## Discussion

In this study we introduced a model where systemic challenge with live *E. coli* induces microglial activation 3 days after inoculation, but this activation did not result in production of pro-inflammatory cytokines in the brain. We found more vigorous microglial activation after a single systemic challenge of LPS, with an increase of pro-inflammatory mediators in the brain 2 days after inoculation.

These observed differences in microglial activation and inflammatory response in the brain is in line with previously reported findings (Hoogland et al., 2015), where we suggested distinct differences in microglial activation between systemic stimulation with (supranatural doses) LPS and live or heat-killed bacteria, although heterogeneity among the included studies precluded a firm conclusion. Exact comparison of the dose of LPS (5 mg/kg) with the dose of *E. coli* (1 × 10^4^ CFU) used in our experiments is very difficult. However, according to a rough approximation (where 5 × 10^6^ endotoxin units (EU)/ml corresponds to 5 mg/kg and 1 EU is generated by 1 × 10^5^ CFU's) about 1.25 × 10^11^ CFU are required to yield such an amount of LPS, suggesting that the suspension *E. coli* we used contained about 1 × 10^7^ less CFU's than the LPS suspension. In only one previous study, by Puntener et al. live bacteria were used to systemically infect adult mice and the effects on microglial cells were examined (Puntener et al., 2012). Many studies included in the systematic review used systemic LPS and reported significant microglial activation (Hoogland et al., 2015), whereas the study where mice were inoculated with 1 × 10^6^ CFU's *Salmonella typhimurium* SL3261 only found moderate microglial activation 7 days after inoculation, showing increased expression of CD11b and CD68 cells in the thalamus (Puntener et al., 2012). With regard to inflammatory response in brain tissue, studies using LPS showed increased pro-inflammatory mediators (such as TNF-α, IL-1β, and IL-6) in the brain, whereas in the study using live bacteria, brain levels of IL-1β were not increased at the time of moderate microglial activation.

Compared to the study by Puntener et al. (2012), where moderate microglial activation was found 7 days after inoculation, we report more profound microglial activation occurring relatively early (3 days) after systemic infection challenge. The differences between studies may well be explained by different disease severity between disease models. Our *E. coli* mouse model would be fatal if the mice were not treated with ceftriaxone. However, comparable to Puntener et al. (2012), there were no quantifiable differences in pro- or anti-inflammatory mediators between infected and control groups in brain homogenate when microglial activation was present. We propose three possible explanations. First, it could be a detection problem. The deltaCt's of the measured inflammatory mediators in brain homogenate are low, preventing the detection of small differences between control and infected groups. Second, the inflammatory response may be region specific. At 48 h after *E. coli* inoculation, we observed microglial activation in cortex but not in hippocampus, thalamus and caudate nucleus/putamen. Finally, the defined microglial activation at later time points might not be real activation but priming of microglial cells. Like activated microglia, primed microglial cells are increased in cell size, increase the expression of activation markers on their cell surface, and proliferate. However, primed microglial cells are morphologically more ramified than activated microglial cells and do not produce inflammatory mediators (Perry and Holmes, 2014).

Conversely, in LPS injected mice, brain mRNA expression levels of TNF-α, IL-1β, IL-6, MCP-1, and M-CSF in brain homogenates were elevated at the 3 h time point as compared to controls, while all microglia activation parameters were negative, questioning where these cytokines come from if they are not induced by activated microglial cells. Qin et al. observed an elevation of TNF-α mRNA and protein levels in brain only 30 min after a single systemic LPS challenge (5 mg/kg), with a peak at 1 h (Qin et al., 2007). Their findings suggest that systemic LPS acts by inducing systemic TNF-α synthesis, which is then transported across the blood brain barrier via TNF-α receptors and activates microglial cells to induce TNF-α and other cytokines (Qin et al., 2007). When looking at our data, in the LPS 3 h group, almost all cytokines that are upregulated in brain (TNF-α, IL-1β, IL-6, MCP-1, and M-CSF) are also upregulated in spleen (IL-1β, IL-6, and M-CSF), which partly confirms the finding of Qin et al. that peripheral cytokines are possibly transported over the blood brain barrier after systemic challenge with LPS (Qin et al., 2007).

HMGB1, a protein previously known only as a nuclear transcription factor, has been implicated as a mediator of delayed endotoxin lethality in systemic inflammation (Andersson and Tracey, 2003). In our study mRNA expression of HMGB1 was down regulated after LPS stimulation and up regulated after infection with *E. coli*, in spleen homogenate in more extent than in brain, which implicates a different course of inflammatory response. Differences of HMGB1 expression in the two models may also be explained by difference in time points. However, HMGB1 has also been suggested to mediate neuro-inflammatory priming in the aged brain and HMGB1 antagonism prevented prolonged infection-induced neuro-inflammatory and sickness responses in aged rats treated with live *E. coli* (Fonken et al., 2016).

### Limitations

To monitor the effect of ceftriaxone as a third variable we included an extra control group where ceftriaxone was added. No differences in neuro-inflammatory response was found compared to the control group where saline was added. To examine the effect of ceftriaxone during inflammation, ceftriaxone has to be added to the LPS groups. Our main goal was to introduce a mouse model where systemic challenge with live bacteria causes a neuro-inflammatory response, therefore to fully examine the effect of ceftriaxone was beyond the scope of our study.

The protocol for isolating and staining microglial cells for flow cytometry was based on a human protocol (Melief et al., 2012). In this human protocol, intermediate CD45 expression distinguished microglial cells from infiltrating macrophages, which had high CD45 expression. In our mouse samples there was only one cell population of CD45 positive cells. When this population was compared to a blood sample which was stained with CD45, the expression of CD45 positive cells of the brain samples was much lower. Furthermore, before brain tissue was collected, the brain was perfused with PBS via the left ventricle of the heart, preventing interference of blood cells. Therefore, we concluded that this one cell population of CD45 positive cells were in fact all microglial cells.

The next step in research is to implement this *E. coli* model in different experiments. We used young adult mice, while sepsis associated delirium and its late complications, is most common in older patients (Inouye, 2006). It is well-known that aging has its effect on the adaptive immune system. The innate immune system remains intact whereas the adaptive immune system reduces its activity, resulting in a chronic low-grade pro-inflammatory state, also known as inflamm-aging (Cevenini et al., 2013). This suggests an important role of aging in the process of connecting systemic inflammation, microglial activation, and sepsis associated delirium. The introduction of this model is a first step; although an extreme phenotype of microglia activation was not found, this finding is in line with our hypothesis (Witlox et al., 2010). Future experiments should evaluate differences in microglial response after infection with live bacteria in old and young mice.

## Conclusion

We conclude that it is possible to cause microglia activation with an abdominal infection model with live *E. coli*. We observed distinct differences in microglial activation between systemic stimulation with live *E. coli* and (supranatural doses of) LPS, with earlier, more profound microglial activation and inflammatory response in the brain after systemic LPS. Because the animal model, where live bacteria are used to cause an infection and this infection is subsequently treated with antibiotics, simulates the clinical situation closely, it might be more suited to examine the pathogenesis of infection associated delirium than the LPS model.

## Availability of data and materials

The datasets used and/or analyzed during the current study are available from the corresponding author on reasonable request.

## Author contributions

The work presented was carried out in collaboration between all authors. Experimental procedures were carried out by ICMH, DW, and MV with technical assistance of JH-W. The protocol for isolated microglia for flow cytometry was provided by JM and IH. Flow cytometric analysis was performed by ICMH with assistance of JM. Histopathological assessment of slides was performed by ICMH and J-YE-L. The manuscript was drafted by ICMH and discussed and edited by DvdB. All authors have read and approved the final version of the manuscript.

### Conflict of interest statement

The authors declare that the research was conducted in the absence of any commercial or financial relationships that could be construed as a potential conflict of interest.

## Abbreviations

7-AAD: 7-amino-actinomycin D
APC: allophycocyanin
CD11b: cluster of differentiation 11b
CD16: cluster of differentiation 16
CD32: cluster of differentiation 32
CD45: cluster of differentiation 45
cDNA: complementary deoxyribonucleic acid
CFU's: colony-forming units
CI: confidence interval
CNS: central nervous system
CSF: cerebral spinal fluid
DAB: diaminobenzidine
deltaCt: delta cycle threshold
*E. coli*: *Escherichia coli*
EU: endotoxin units
GKN-BSA: glucose-potassium-sodium buffer
HMGB1: high-mobility group 1
Iba-1: ionized calcium binding adaptor molecule 1
Ig: immunoglobulin
IL-1β: interleukin 1 beta
IL-6: interleukin 6
IL-12: interleukin 12
IVC: individually ventilated cages
LB: Luria-Bertani medium
LPS: lipopolysaccharide (LPS)
MCP-1: monocyte chemotactic protein 1
M-CSF: macrophage colony-stimulating factor
mRNA: messenger ribonucleic acid
NoNo: Non-POU-domain containing octamer binding protein
PBS: phosphate buffered saline
PE: phycoerythrin
PerCP: peridinin-chlorophyll-protein
qPCR: quantitative polymerase chain reaction
RNA: ribonucleic acid
TGF-β: transforming growth factor beta
TNF- α: tumor necrosis factor alpha.
