# Supplementary material for: Microglial Activation After Systemic Stimulation With Lipopolysaccharide and Escherichia coli
Source: Front Cell Neurosci. 2018 Apr 24;12:110. doi: 10.3389/fncel.2018.00110 (PMC5932388; doi:10.3389/fncel.2018.00110)
Supplement: Supplementary file 1 [file DataSheet_1.doc]

**APPENDIX**

|  |  |  | Number of mice per experimental group | | | |
| --- | --- | --- | --- | --- | --- | --- |
| Round | Challenge | Time point | NaCl | NaCl+Ceft | LPS/*E. coli* | *E. coli*+Ceft |
| 1 | *E. coli* | t=12h | 6 |  | 15 |  |
| 2 | *E. coli* | t=20h | 6 |  | 15 |  |
| 3 | *E. coli* | t=48h | 6 | 6 |  | 15 |
|  |  | t=72h | 6 | 6 |  | 15 |
| 4 | LPS | t=3h | 6 |  | 15 |  |
|  |  | t=48h | 6 |  | 15 |  |

Table 1: Group size per experiment and time point. Ceft=Ceftriaxone.

| Gene | Direction | PCR Primer 5' 🡪 3' | Product size (bp) |
| --- | --- | --- | --- |
| NoNo | Forward | TGCTCCTGTGCCACCTGGTACTC | 170 |
|  | Reverse | CCGGAGCTGGACGGTTGAATGC |  |
| TNF-α | Forward | ACGGCATGGATCTCAAAGAC | 138 |
|  | Reverse | AGATAGCAAATCGGCTGACG |  |
| IL-1β | Forward | GGGCCTCAAAGGAAAGAATC | 183 |
|  | Reverse | TACCAGTTGGGGAACTCTGC |  |
| IL-6 | Forward | AGTTGCCTTCTTGGGACTGA | 191 |
|  | Reverse | CAGAATTGCCATTGCACAAC |  |
| HMGB1 | Forward | CCATTGGTGATGTTGCAAAG | 158 |
|  | Reverse | CTTTTTCGCTGCATCAGGTT |  |
| M-CSF | Forward | CCTGTGTCCGAACTTTCCAT | 181 |
|  | Reverse | TACAGGCAGTTGCAATCAGG |  |
| MCP-1 | Forward | AGCACCAGCCAACTCTCACT | 185 |
|  | Reverse | TCATTGGGATCATCTTGCTG |  |
| TGF-β | Forward | TTGCTTCAGCTCCACAGAGA | 183 |
|  | Reverse | TGGTTGTAGAGGGCAAGGAC |  |
| IL-12 | Forward | CATCGATGAGCTGATGCAGT | 163 |
|  | Reverse | CAGATAGCCCATCACCCTGT |  |

Table 2: Primer sequences.

Figure 1: Weight difference in percentage.

Figure 2: Bacterial outgrowth in cerebral spinal fluid (CSF), blood, spleen and liver per time point. Note that mice at the 48 hour and 72 hour time points received ceftriaxone at 12 hours and 24 hours after inoculation. Data represent mean + SEM. The red dotted line is the lower limit of detection (LLD), LLD for CSF is 2000 CFU/ml, LDD for blood is 20 CFU/ml and LDD for spleen and liver is 100 CFU/ml.


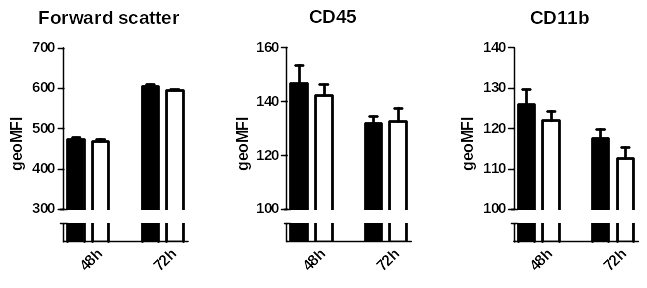

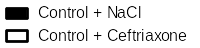


Figure 3: Geometric means (geoMFI) for forward scatter, expression of cluster of differentiation 45 (CD45) and CD11b measured with flow cytometry for control groups with additional saline or Ceftriaxone. Data represent mean + SEM, *P<0.05, **P<0.01, ***P<0.001. NB: Flow cytometry for every time point was done on a different day. Laser characteristics vary per day, therefore the geoMFI’s are not comparable between experiments. Hence every group has its own control group.
